# Features and Limitations of Robotically Assisted Percutaneous Coronary Intervention (R-PCI): A Systematic Review of R-PCI

**DOI:** 10.3390/jcm13185537

**Published:** 2024-09-19

**Authors:** Max Wagener, Yoshinobu Onuma, Ruth Sharif, Eileen Coen, William Wijns, Faisal Sharif

**Affiliations:** 1 College of Medicine, Nursing and Health Sciences, University of Galway, H91 TK33 Galway, Ireland; 2 Department of Cardiology, University Hospital Galway, Newcastle Road, H91 YR71 Galway, Ireland; 3 CORRIB Research Centre for Advanced Imaging and Core laboratory, H91 TK33 Galway, Ireland

**Keywords:** robotically assistedPCI, R-PCI, PCI, percutaneous coronary intervention

## Abstract

**Background:** Ischaemic heart disease is one of the major drivers of cardiovascular death in Europe. Since the first percutaneous coronary intervention (PCI) in 1977, developments and innovations in cardiology have made PCI the treatment of choice for stenotic coronary artery disease. To address the occupational hazards related to chronic exposure to radiation and wear and tear from heavy lead-based radioprotective aprons, the concept of robotically assisted PCI (R-PCI) was introduced in 2005. **Aim:** To explore the features and limitations of R-PCI, we first discuss the concept and evolution of R-PCI platforms and then systematically review the available clinical data. **Methods:** A systematic review has been performed across the Pubmed, Embase and Cochrane databases in order to assess the efficacy and safety of R-PCI. Secondary endpoints, such as operator and patient exposure to radiation, contrast volume used and procedural time, were assessed when available. **Results:** In selected patients, R-PCI provides high technical and clinical success rates, ranging from 81 to 98.8% and from 93.3 to 100%, respectively. In-hospital and 1-year MACE rates ranged from 0 to 10.4% and 4.8 to 10.5%, respectively. R-PCI is able to significantly reduce the operator’s exposure to radiation. Further research analysing the patient’s and cath lab staff’s exposure to radiation is needed. Therapy escalation with R-PCI seems to be limited to complex lesions. R-PCI procedures add approximately 10 min to the procedural time. **Conclusions:** The efficacy and safety of R-PCI have been proven, and R-PCI is able to significantly reduce occupational hazards for the first operator. The lack of adoption in the community of interventional cardiologists may be explained by the fact that current generations of R-PCI platforms are limited by their incompatibility with advanced interventional devices and techniques needed for escalation in complex interventions.

## 1. Introduction

Being responsible for 3.8 million deaths yearly, cardiovascular disease is the leading cause of death in Europe, and ischaemic heart disease, as one of the major drivers, accounts for 47% of all cardiovascular deaths [1]. Since the first percutaneous coronary intervention (PCI) by Andreas Grüntzig in 1977, need- and innovation-driven evolution in the field has made minimally invasive catheter-based interventions the treatment of choice for stenotic coronary artery disease [2,3]. From a patient’s view, novel techniques and devices should meet or exceed the efficacy and safety standards of the current state-of-the-art practice in manual PCI [4,5,6,7]. Compatibility with current and future generations of interventional cardiologists’ armamentarium is essential for robotically assisted PCI (R-PCI) [3,8].

Considering occupational hazards among cardiac catheterisation laboratory (cath lab) workers, up to 19.5% suffer from orthopaedic problems related to heavy lead-based radioprotective aprons [9]. Of greater concern are the deterministic and stochastic effects resulting from chronic exposure to ionising radiation [10]. Despite improved radioprotection measures over the past few years, the potential risk of cancer attributable to work-related radiation exposure cannot be denied [11,12]. In addition to the risk of left-sided brain malignancies, blood count alterations have been associated with occupational radiation exposure [9,13,14]. Furthermore, significantly higher rates of posterior lens opacities have been described in interventional cardiologists in comparison to a non-exposed control group, with a relative risk (RR) of 5.7 (95%CI 1.5–22) [15].

With the promise of lowering occupational hazards for interventional cardiologists, R-PCI was introduced in 2005 [6,16]. While robotic assistance is currently established in the field of surgery, scepticism in the community of interventional cardiologists has limited its adoption in daily practice [6,16,17]. To explore the features and limitations of R-PCI, we first discuss the concept and evolution of R-PCI platforms and then systematically review the available clinical data [18,19].

## 2. Methods

To set the scientific context, we first discuss the concept and evolution of R-PCI. **Systematic search strategy**—We systematically searched the Pubmed, Cochrane and Embase databases using (*robotically assisted percutaneous coronary intervention*) *OR* (*robotically assisted PCI*) *OR* (*R-PCI*) as a search strategy. **Inclusion**—Original research papers were considered for this systematic review. **Exclusion**—In order to avoid double inclusions of identical patient cohorts, pooled-data analyses and meta-analyses were excluded. In addition, animal studies, conference abstracts, study protocols, case reports and case series (*n* < 15) were excluded. In the second step, redundant search results across the different databases were eliminated. **Statistics**—With the lack of unified endpoint definitions and in the context of heterogeneous patient cohorts, we did not conduct a meta-analysis, and efficacy and safety endpoints were consolidated in a descriptive approach. Secondary endpoints, such as operator and patient exposure to radiation, contrast volume used and procedural times, were assessed when available.

## 3. Results

**Figure 1:**
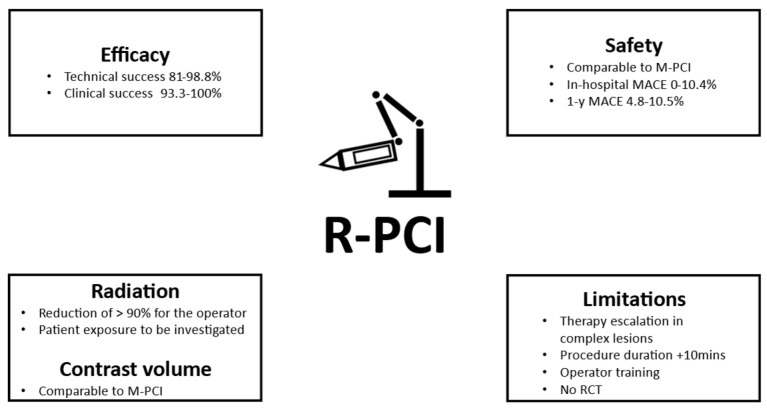
Essential Findings. (MACE = major cardiovascular event; M-PCI = manual percutaneous coronary intervention; R-PCI = robotically assisted percutaneous coronary intervention; y = year.

The essential findings of the systematic review, as elaborated below, are summarised in Figure 1.

### 3.1. Concept and Evolution of R-PCI

The concept of remotely performing PCI was introduced in 2005 with the **remote navigation system (RNS)** (NaviCath, Haifa, Israel) [16]. It had a dual navigator system, the first allowing continuous or stepwise wire manipulations with 2 degrees of freedom (axial and rotational) and the second for device (balloon/stent) manipulation, allowing axial movement via a set of rollers [16]. After in vitro and animal proof-of-concept series, a first-in-human pilot study to prove feasibility and safety in 18 patients was published in 2006 [6,16].

Further development led to the next more user-friendly device, the **Corindus Corpath 200** (Corindus, Waltham, MA, USA), providing single-use cassettes that contained both navigation units for wire and device manipulation [20].

**Figure 2:**
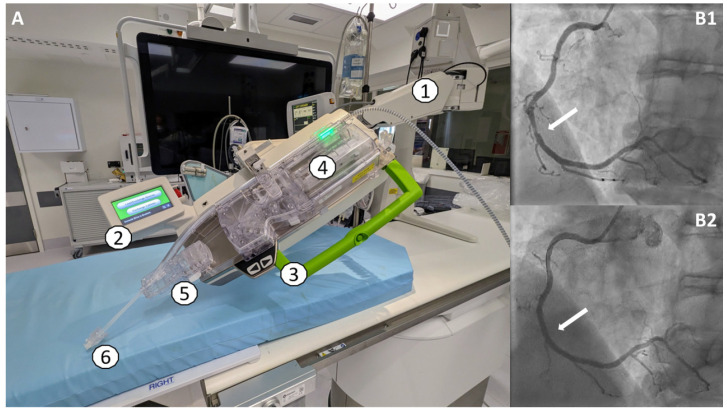
(**A**)—The second-generation R-PCI system, Corindus CorPath GRX, at University Hospital Galway. (1) The articulated robotic arm, directly connected to the PCI table. (2) The touch screen. (3) The robotic drive to which the single-use cassette (4) is attached. (5) An additional drive for the Y-connector/guide catheter. (6) The sheath connector. (**B1**,**B2**)—The first patient undergoing R-PCI in Ireland and the UK on 12 December 2022. The right coronary artery before (**B1**) and after (**B2**) R-PCI. The white arrows indicate the coronary lesion before and after treatment.

Whereas the first generation, Corindus CorPath 200, was limited to the robotic control of guidewires and devices (PCI balloons and stents), the second generation, **Corindus CorPath GRX** (Corindus/Siemens Healthineers, Waltham, MA, USA), introduced in 2018, provided an additional drive, allowing robotically assisted guide catheter movements in two dimensions (axial and rotational) (Figure 2) [7]. Furthermore, software development introduced automated algorithms for assisted wire manipulation, providing rotation-on-retraction, wiggle and spinning functions [7].

In parallel to the Corindus platforms, the **R-One robotic system** (Robocath, Rouen, France) was CE-marked in Europe in 2019 [21]. Following the same principles, the system is built around two components: a bedside-mounted robotic arm with an attachment for the single-use cassette and a radio-protected control station [21]. Similar to the Corindus CorPath 200, two tracks are available: one for guidewire manipulation with two degrees of freedom (translational and rotational) and a second track for the manipulation of PCI balloons and stents [21]. In contrast to the second-generation Corindus CorPath GRX (Corindus/Siemens Healthineers, Waltham, MA, USA), this first iteration of the R-One platform does not provide an additional drive for active catheter manipulation, and to date, the software has not integrated automated algorithms for wire manipulation [7,21].

When sitting just in front of a dedicated screen on the control console, the first operator’s visual perception of the fluoroscopic and cine acquisitions is enhanced. As R-PCI allows for the axial positioning of guidewires, balloons and stents in 1 mm increments, R-PCI conceptually has the potential to augment precision in treatment. Thus, R-PCI has the potential to lower the risk of geographic miss and to decrease the length of stents implanted, both known to be associated with the risk of stent failure [22].

### 3.2. Systematic Search Results

The systematic search led to 214, 80 and 25 results on Pubmed, Embase and the Cochrane Library, respectively. After the consideration of inclusion and exclusion criteria and the elimination of redundant search results/patient cohorts, 29 publications were considered for this manuscript. The last search was performed on 27 July 2024 during manuscript revision (Supplementary Figure S1—Decision flowchart).

### 3.3. Efficacy

**Figure 3:**
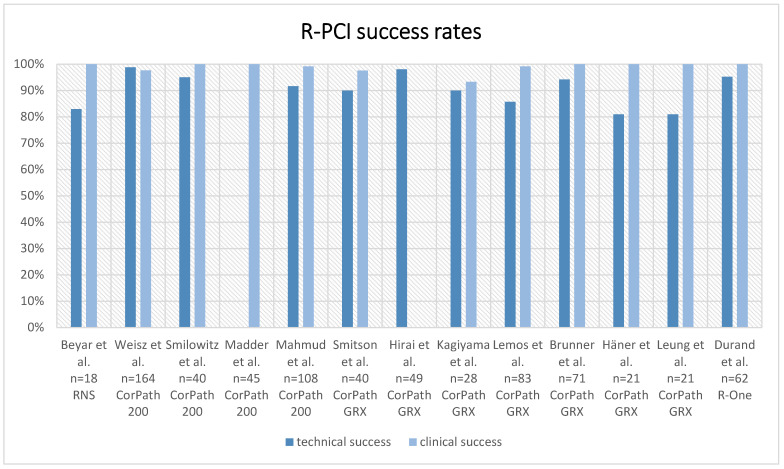
Technical and clinical success rates across different trials and R-PCI platforms. Technical and clinical successes are per individual trial definitions (see Table 1). Madder et al. and Hirai et al. did not report the technical or clinical success rate as a separate endpoint [4,6,7,21,24,25,26,27,28,29,30,31,32].

To date, unified definitions of technical or clinical success rates of R-PCI are lacking. A commonly used criterion of *technical success* is whether or not a procedure was robotically assisted from the beginning to the end of the procedure [23]. *Clinical success* in the context of R-PCI is commonly defined by whether the intended lesion was successfully treated without the occurrence of a major cardiovascular event (MACE) [23]. Table 1 summarises the definitions of efficacy endpoints as well as the results across different R-PCI platforms. Figure 3 displays efficacy endpoints across different trials.

Using the **RNS system** (NaviCath, Haifa, Israel), Beyar et al. reported high technical (83%) and clinical (100%) success rates [6]. In one patient, guidewire navigation was unsuccessful due to technical problems with the RNS, and in two cases, stent deployment was performed manually due to a system malfunction [6].

With data available from four trials covering 357 patients undergoing R-PCI using the first-generation **Corindus CorPath 200** platform (Corindus, Waltham, MA, USA), technical and clinical success rates ranged from 91.7% to 98.8% and 97.6% to 100%, respectively [4,24,25,26].

Transitioning to the second-generation **Corindus Corpath GRX** platform (Corindus/Siemens Healthineers, Waltham, MA, USA), Smitson et al. reported similarly high technical and clinical success rates in their single-arm cohort of 40 patients with a high proportion of type B2/C lesions (77.8%) [7]. Technical success was achieved in 90% of patients [7]. One chronic total occlusion (CTO) could not be crossed either robotically or manually [7]. One case needed a buddy wire to cross a stent through the struts of a previously placed stent [7]. In another case, wire crossing of a highly stenosed (95%), long-segment (25 mm) type C lesion was not possible robotically, and the authors assumed a suboptimal grip of the wire in the robotic drive cassette [7]. A third balloon-uncrossable lesion needed unplanned advanced plaque modification via orbital atherectomy [7]. All in all, a clinical success rate of 97.5% was achieved in this cohort [7].

Hirai et al. assessed the use of R-PCI (with second-generation Corindus CorPath GRX platform (Corindus/Siemens Healthineers, Waltham, MA, USA)) in a retrospective study of patients who underwent successful PCI for a single **CTO lesion** [27]. Patients were treated following a hybrid approach, and after manual lesion crossing (including the use of microcatheters, atherectomy devices and over-the-wire CTO-PCI devices), the guidewire was replaced by a workhorse wire, and the guide catheter was connected to the R-PCI platform [27]. Ninety-eight percent of the planned R-PCI cases were resolved according to the prespecified hybrid protocol [27]. In one patient, an unplanned interruption was mandated due to acute thrombus formation that required intracoronary tPA application [27]. Notably, patients in the R-PCI and M-PCI groups had comparable target lesion complexity, with a J-CTO Score of 2.1 ± 1.1 in the R-PCI group and 2.0 ± 1.2 in the M-PCI group, *p* = 0.81 [27].

When including data from five additional single-centre studies (*n* = 313) using the CorPath GRX platform, technical and clinical success rates range from 81% to 98% and 93.3% to 100%, respectively [28,29,30,31,32].

The European multicentre prospective R-EVOLUTION study assessed clinical and technical success rates in patients undergoing R-PCI using the **Robocath R-one** system (Robocath, Rouen, France) [21]. Sixty-two patients with de novo coronary artery stenosis and an ACC/AHA lesion type B2/C proportion of 25% (16 of 64 lesions) underwent R-PCI using radial access in most cases (96.8%) [21]. Clinical success was achieved in all patients (100%) [21]. Technical success was achieved in 95.2% [21].

In all of the above-mentioned trials, the reasons for manual conversion or partial manual assistance were of similar origin and, among other factors, related to poor guide catheter support, a lack of back-up and/or resistance to balloon/stent delivery. Complex lesions requiring therapy escalation, such as guide extension catheters, buddy wires or orbital atherectomy, were other reasons for conversion [4,5,7,24,25,26,27,28,29,30,31,32]. Conversions for adverse events seem proportionally rare, and technical limitations of the robotic platform were prevalent [4,34]. The detailed reasons for manual conversion are summarised in Table 2.

### 3.4. Safety

**Figure 4:**
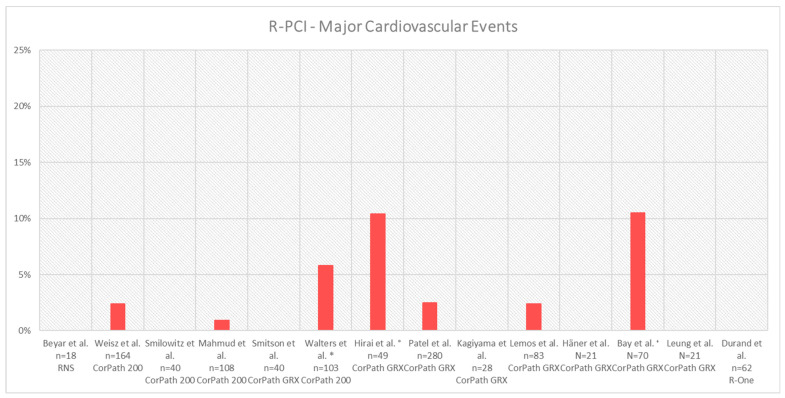
Major cardiovascular event rates in percent across different trials and R-PCI platforms. * MACEs at 6 months; ° CTO cohort that underwent a hybrid approach intervention (manual wire crossing, then change to R-PCI after exchange for workhorse wire). ^†^ MACEs at 12 months (RNS = remote navigation system; R-PCI = robotically assisted percutaneous coronary intervention). [4,5,6,7,21,24,25,27,28,29,30,32,33,35].

Safety endpoints across different trials and R-PCI platforms are summarised in Table 3. Besides minor differences in definition, most trials assessed major cardiovascular events (MACE) as a safety endpoint. Figure 4 summarises the MACE rates across different trials and R-PCI platforms.

Although most trials included highly selected patients, procedural safety seems to be a given. Beyar et al. reported a single non-target lesion myocardial infarction 3 months after the index procedure [6].

The single-arm PRECISE study reported non-Q-wave myocardial infarctions (defined as CK-MB > 3 times the ULN, in the absence of new Q-waves) in 4 (2.4%) out of 164 patients post-R-PCI, none of which had clinical consequences, and no system-related complications or other MACE were reported [24].

In the CORA-PCI study, comparing 108 R-PCI cases to 226 M-PCI cases, the MACE (death, myocardial infarction, stroke, urgent revascularisation) rates were comparable, 0.9% in both groups (*p* = non-significant), and consisted of two clinically significant periprocedural myocardial infarctions in the M-PCI group and one in the R-PCI group [4]. The proportion of procedural CK-MB elevations > 3 times the upper limit of normal (ULN) was not different between groups (R-PCI 5.6%, M-PCI 7.5%, *p* = 0.51) [4].

Walters et al. analysed MACE at 12 months as a primary endpoint in their non-randomised trial using the CorPath 200 system (R-PCI *n* = 103, M-PCI *n* = 210) [33]. At 6 and 12 months of follow-up, there was no significant difference in MACE between groups (MACE 6 months: R-PCI 5.8% vs. M-PCI 3.3%, *p* = 0.51; MACE 12 months (primary endpoint): R-PCI 7.8% vs. M-PCI 8.1%, *p* = 0.92) [33]. No significant difference was seen in the individual variables of the composite endpoint (all-cause mortality R-PCI 2 (1.9%) vs. M-PCI 4 (1.9%), *p* = 0.98; cerebrovascular accident R-PCI 2 (1.9%) vs. M-PCI 3 (1.4%), *p* = 0.73; myocardial infarction R-PCI 3 (2.9%) vs. M-PCI 6 (2.9%), *p* = 0.98; target vessel revascularisation R-PCI 1 (1.0%) vs. M-PCI 4 (1.9%), *p* = 0.54) [33]. Despite a relatively low proportion of radial approaches in both groups (R-PCI 12.6%, M-PCI 13.3%), there were no access-site-related complications meeting the Bleeding Academic Research Consortium (BARC) III criteria [33].

Reporting on a first-in-human single-arm cohort treated with the second-generation Corindus CorPath GRX platform, Smitson et al. reported no in-hospital MACE in 40 patients [7].

In their propensity-score-matched analysis, leaving 280 patients in each group (M-PCI and R-PCI), Patel et al. found similar MACE (composite of target vessel revascularisation, nonfatal myocardial infarction (MI) defined as post-PCI CK-MB (creatine kinase-MB isoenzyme) level of >3× the upper limit of normal or clinical presentation with MI, and death) rates at 30 days [5].

In their retrospective analysis of patients with single CTO lesions (R-PCI *n* = 49, M-PCI *n* = 46), Hirai et al. described comparable MACE rates (R-PCI: 5 (10.4%), M-PCI: 6 (13.0%), *p* = 067) [27], with MACE defined as death, myocardial infarction, clinical perforation, significant vessel dissection, arrhythmia, and acute thrombus and stroke [27]. Of these events, there were two coronary artery perforations in each group (R-PCI 4.1%, M-PCI 4.3%, *p* = not significant). Furthermore, two vessel dissections and one acute vessel thrombosis occurred in the R-PCI group [27]. In the manual group, there were one myocardial infarction, one arrhythmia, one vessel dissection and one acute vessel thrombosis in addition to the above-mentioned perforations [27]. Hirai et al. reported conversion times from R-PCI to M-PCI using the second-generation CorPath GRX platform of less than 1 min [27].

Kagiyama et al. reported no MACE within 72 h or MACE up to hospital discharge in their patients undergoing R-PCI (*n* = 28) after the introduction of the CorPath GRX platform in their centre [29].

Lemos et al. reported an occurrence of the predefined safety endpoint (death or target-vessel-related complication) in 2.4% of patients with one myocardial infarction and one repeat intervention. Not included in the composite endpoint was one in-hospital stroke. At 30 days, one death possibly related to acute stent thrombosis had occurred [30].

Häner et al. reported no in-hospital MACE, and one patient presented with a non-target vessel myocardial infarction within the 12-month follow-up period [32].

The INTERCATH study compared 12-month MACE rates and all-cause mortality in 70 patients with chronic coronary syndromes and non-ST-segment elevation myocardial infarction undergoing R-PCI, matched to 210 patients undergoing M-PCI [35]. There were no significant differences in 12-month MACE (R-PCI 10.5%, M-PCI 6.5%, *p* = 0.25) or all-cause mortality (R-PCI 9.6%, M-PCI 4.8%, *p* = 0.22), and individual MACE components (cardiovascular death, unplanned target lesion revascularisation, nonfatal myocardial infarction and nonfatal stroke) did not differ between groups [35].

Leung et al. reported no in-hospital MACEs, though 1 of 21 patients presented with type 2 myocardial ischaemia in the context of a gastro-intestinal bleed at a later point of time [28].

Analysing the safety and efficacy of the Robocath R-One platform, Durand et al. described an equally high safety profile, with no in-hospital or 30-day MACE reported in their single-arm cohort of 62 patients undergoing R-PCI. A single non-occlusive coronary dissection (NHLBI type B) was evaluated not to be associated with the use of the robotic platform and was treated manually with a second stent [21].

### 3.5. Treatment Precision

Although not assessed as a primary endpoint, in their analysis comparing 40 R-PCI to 80 M-PCI cases, Smilowitz et al. reported no differences in the mean number of stents used per patient (both 1.2 ± 0.4; *p* = 0.76), and stent diameters and lengths were comparable [25].

Bezerra et al. assessed the impact of R-PCI on the incidence of longitudinal geographic miss, which is known to be associated with target vessel revascularisation (6.1% vs. 2.6%, *p* < 0.05) and myocardial infarction (2.4% vs. 0.8%, *p* = 0.04) in comparison to patients without longitudinal geographic miss [36]. The authors performed a propensity-score-matched analysis, matching patients who underwent M-PCI within the STLLR trial to the R-PCI cohort (*n* = 164, CorPath 200) of the PRECISE study [36]. In the unmatched cohort analysis, overall longitudinal geographic miss occurred in 20 of 164 (12.2%) R-PCI patients and in 650 of 1509 (43.1%) M-PCI patients [36]. In the matched group analysis (39 patients per group), longitudinal miss was significantly lower in patients treated with R-PCI (4/39 patients, 10.3%) in comparison to patients in the M-PCI group (25/39 patients, 64.1%) (*p* < 0.0001) [36].

### 3.6. Exposure to Radiation

#### 3.6.1. Operator Exposure to Radiation

Madder et al. prospectively assessed operator exposure to radiation across three groups: M-PCI with conventional lead aprons, M-PCI with suspended lead suits and R-PCI (Corindus CorPath 200) in combination with suspended lead suits [26]. The assessment across 336 PCI cases (R-PCI 13.4% of all cases) revealed significantly reduced radiation exposure using suspended lead suits (Zero-Gravity, CFI Medical, Fenton, MI, USA) in comparison to conventional lead aprons (head exposure 0.5 [1.9] µSv vs. 14.9 [51.5] µSv, *p* < 0.001; chest exposure 0.0 [0.1] µSv vs. 0.4 [4.0] µSv, *p* < 0.001) [26]. In this single-centre analysis, R-PCI in combination with suspended lead suits was associated with the lowest radiation exposure for the operator. Chest exposure in this group was reduced to 0.0 [0.0] µSv, being significantly lower in comparison to the two comparators [26]. Similarly, head exposure was 0.1 [0.2] µSv, significantly lower [26]. Thus, R-PCI in combination with suspended lead suits allowed a 99.3% reduction in head level radiation exposure in comparison to conventional lead aprons used for manual PCI and an 80.0% reduction in comparison to suspended lead suits [26].

Using the Robocath R-One platform, Durand et al. assessed radiation exposure on and under the lead apron in a single cohort of 62 patients [21]. For comparison, a simulated manual operator model was used, placing a pole with dosimeters on and under a lead apron in typical position of a manual operator [21]. Operator radiation exposure could be reduced from 57.1 (±61.2) µSv to 7.2 (±8.7) µSv on the lead apron and from 3.2 (±4.1) µSv to 0.2 (±0.5) µSv underneath the lead apron [21]. The overall radiation dose was reduced by 77.1 (±26.1)% on the lead apron and by 84.5 (±25.2)% under the lead apron [21].

#### 3.6.2. Patient Exposure to Radiation

Beyar et al. compared total fluoroscopy time (TFT) in 18 patients undergoing R-PCI to a non-randomised control group of 20 consecutive patients undergoing manual, single-lesion PCI and found no significant differences between groups. (R-PCI 8.8 ± 4.8 min, M-PCI 9.1 ± 3.5 min, *p* = ns) [6].

Smilowitz et al. reported a trend towards a lower TFT (R-PCI (*n* = 40) 10.1 ± 4.7 min, M-PCI (*n* = 80) 12.3 ± 7.6 min, *p* = 0.05) but no statistically significant difference in the total radiation dose (R-PCI 1389 ± 599 mGy, M-PCI 1665 ± 1026 mGy, *p* = 0.07) [25].

The CORA-PCI-study showed comparable fluoroscopy times in both groups (R-PCI 18.2 ± 10.4 min, M-PCI 19.2 ± 11.4 min, *p* = 0.39), and a significant difference in the dose area product (R-PCI 12,518 ± 15,970 cGycm^2^ M-PCI 14,048 ± 18,437 cGycm^2^, *p* = 0.045) was later confirmed not to be significant after propensity score matching [4].

In the CTO-PCI analysis by Hirai et al., significantly shorter fluoroscopy times were noted: 37.9 ± 17.9 min in the R-PCI group vs. 48.6 ± 17.1 min in the M-PCI group (*p* < 0.01) [27]. The radiation dose assessed in air kerma was significantly lower in the R-PCI group (1522 ± 1129 mGy vs. 2466 ± 1204 mGy, *p* < 0.01) [27].

One of the largest trials examining patient radiation exposure as a co-primary endpoint (air kerma, dose area product, fluoroscopy time, volume of contrast and total procedural time) was led by Patel et al., looking at a total of 996 consecutive patients in a propensity-score-matched analysis [5]. Of these patients, 310 (31.1%) patients underwent R-PCI, and 686 (68.9%) patients underwent M-PCI [5]. Propensity score matching considered, among other things, lesion complexity, as assessed by SYNTAX-Score [5], leaving 280 patients in each group with a comparable distribution of target lesions and comparable SYNTAX-Scores 14 (10–21) in the M-PCI group and 13 (9–17) in the R-PCI group (*p* = 0.433) [5]. Although the fluoroscopy time was not different across groups, patient radiation exposure was more favourable for R-PCI, with an air kerma of 884 (699–1498) mGy for R-PCI and 1110 (1699–1498) mGy for M-PCI (*p* = 0.002), and similarly for the dose area product, with 4734 (2695–7746) cGy.cm^2^ in the R-PCI group and 5746 (3751–7833) cGy.cm^2^ (*p* = 0.003) in the M-PCI group [5].

The effect of operator training on patient radiation exposure is not deniable. Although not powered for conclusions, Leung et al. described significantly higher radiation doses in their early patients in comparison to the later patients, with dose area products of 967.3 ± 863.2 and 361 ± 231.1, respectively (*p* = 0.01) [28].

### 3.7. Contrast Volume

Smilowitz et al. reported similar volumes of contrast used in patients undergoing R-PCI or M-PCI (121 ± 47 mL vs. 137 ± 62 mL, respectively, *p* = 0.11) [25]. In their study of consecutive patients with comparable target lesions (R-PCI *n* = 45, M-PCI *n* = 123), Madder et al. assessed comparable contrast volumes used in both groups (167 (89) mL in R-PCI and 145 (92) mL in M-PCI, *p* = 0.12) [26]. In the CORA-PCI study, a trend towards a lower contrast volume used in the R-PCI group (183.4 ± 78.7 mL vs. 202.5 ± 74 mL in the M-PCI group, *p* = 0.031) did not remain significant in the propensity-matched analysis [4]. In the context of CTO-PCI using a hybrid R-PCI approach, Hirai et al. reported similar contrast volumes used, 111 ± 39 mL in the R-PCI group vs. 118 ± 53 min in the M-PCI group (*p* = 0.47) [27]. In their propensity-score-matched analysis, Patel et al. reported comparable amounts of contrast used in each group (N = 560 with 1:1 matching) [5], with 130 (103–170) mL being used in the M-PCI group and 140 (100–180) mL in the R-PCI group (*p* = 0.905) [5].

### 3.8. Procedural Times

Limited by a small population size (R-PCI *n* = 18, M-PCI *n* = 20), Beyar et al. reported no significant differences in procedural time in patients undergoing single-lesion PCI (ACC/AHA lesion types A–C) (R-PCI 44 ± 32.7 min, M-PCI 61 ± 19 min, *p* = ns) [6].

Although not assessed as a primary outcome, Madder et al. showed significantly longer procedural times in patients undergoing R-PCI (*n* = 45) in comparison to patients undergoing conventional M-PCI (*n* = 123). With a median of one lesion in comparable locations treated in both groups, the median duration of R-PCI was 55 [22.0] min and 45 [37.0] min in M-PCI (*p* = 0.02) [26].

In the CORA-PCI study, propensity-score-matched subgroup analysis showed significantly longer procedural times in the R-PCI group, 42 min 59 s (±26:14) in comparison to 34 min 01 s (±17:04) in the M-PCI group (*p* = 0.007) [4].

In their propensity-score-matched analysis, Patel et al. reported a significantly longer procedural time in patients undergoing R-PCI (37 (27–50) min) than in patients undergoing M-PCI (27 (21–40) min) (*p* < 0.0005) [5].

In the specific group of patients with CTO lesions, Hirai et al. found comparable overall procedural times, with 89.6 ± 27.1 min in the R-PCI group and 93.4 ± 30.5 min in the M-PCI group (*p* = 0.52) [27]. The time from lesion crossing (manual in both groups, with a switch to R-PCI after crossing) to the removal of the guide catheter after the completion of the intervention was significantly longer in the R-PCI group: 40.6 ± 12.7 min vs. 32.1 ± 17.8 min (*p* < 0.01) [27]. As a percentage of the total procedural time, with 47.8 ± 16.2% in the R-PCI group and 35.5 ± 16.4%, this difference remained significant (*p* < 0.03) [27].

Looking at procedural times with the Robocath R-One platform, Durand et al. compared procedural times in experienced centres (>5 R-PCI procedures prior to patient enrolment) vs. those in early-experience centres [21]. The overall procedural time (time from sheath placement to removal) was significantly shorter in experienced centres, with 35.50 (±11.12) min vs. 45.25 (±16.60) min in less experienced centres (*p* = 0.01) [21]. A non-significant trend was recorded for the robotic duration (first robotic manipulation to last guidewire removal) with 17.47 (±8.02) min vs. 22.23 (±10.99) min (*p* = 0.07) [21]. These findings lead to the assumption that, with growing experience, the time to set up the robotic platform can be reduced. In this study, a comparison to a M-PCI cohort was not assessed.

## 4. Discussion

In selected patients, R-PCI has been shown to have high clinical and technical success rates, ranging from 93.3 to 100% and 81 to 98.8%, respectively. In comparison to M-PCI, R-PCI provides a comparable safety profile. Although randomised controlled data are lacking, in-hospital (0–10.4%) and 1 y MACE (4.8–10.5%) rates, as assessed in the propensity-score-matched analyses, are promising [4,5]. Optimised visualisation has the potential to increase treatment precision and reduce longitudinal geographic miss [36]. R-PCI provides the great benefit of significantly reducing radiation exposure for the first operator and, although not analysed, conceptually has the potential to reduce the risk of orthopaedic damage related to heavy lead aprons [26].

Nonetheless, several limitations of the current generations of R-PCI are worth discussing.

### 4.1. Limited Escalation Features for Complex Interventions

Analysing the reasons for conversion from R-PCI to M-PCI during procedures, uncrossable lesions with a need for escalation to interventional support techniques such as buddy wires and/or guide extension catheters were reported in the trials [7,24,25]. One could also hypothesise that the lack of haptic feedback, which experienced operators rely on, significantly limits current generations of R-PCI platforms, especially in the setting of elevated resistance through tight lesions and tortuous vessels. Conversions to a manual procedure due to limited guide catheter support were reported in CORA-PCI and the R-EVOLUTION study [4,21]. Second-generation platforms, which allow robotically assisted guide catheter manipulations, such as the Corindus CorPath GRX, may elevate the threshold of manual conversion in this matter [4,7,21].

### 4.2. Lack of Compatibility with Intravascular Imaging or Invasive Physiology

To date, none of the R-PCI platforms has regulatory-approved compatibility with intracoronary pressure wires for physiological assessment or intravascular imaging. In complex PCI, intravascular imaging should be considered the standard of care and has been shown to improve patient outcomes; therefore, compatibility should be made available in future iterations of R-PCI platforms [37]. Although Koeda et al., Kimura et al. and Leung et al. have shown the feasibility and safety of intravascular imaging (intravascular ultrasound/optical coherence tomography) in patients undergoing R-PCI, imaging had to be manually performed [28,38,39].

### 4.3. Lesion Crossing in Chronic Total Occlusions

Although it is the most advanced commercially available R-PCI platform, the Corindus Corpath GRX is not compatible with over-the-wire complex coronary intervention devices, such as microcatheters or atherectomy devices for advanced plaque modulation/penetration [27]. Also, the compatibility of the R-PCI platform with rapid-exchange devices other than balloons and stents is limited [27]. Thus, in the CTO cohort studied by Hirai et al. in a per-protocol analysis, all lesions in the R-PCI group were manually crossed, and the R-PCI platform was only connected after the exchange of the (CTO) guidewire for a workhorse wire [27].

### 4.4. Operator Training

Currently, cardiologists undergo specific fellowships to become trained as an interventional cardiologist. Structured programmes around coronary and structural heart interventions are offered at high-volume centres to obtain high standards in training. In the R-EVOLUTION trial with the Robocath R-One platform, significantly longer procedural times in unexperienced centres in comparison to experienced centres were recorded [21]. Comparing early to later cases, Leung et al. reported a statistically significant decrease in fluoroscopy times from 44.0 ± 16.7 min to 25.2 ± 9.1 (*p* = 0.008) [28]. These results suggest the need for specific operator, nurse and technician training programmes.

## 5. Perspectives

### 5.1. Potential for Telemedicine

One of the most appealing and potentially revolutionising features of R-PCI is its potential use in a telemedical setting. This would open the door to treating patients in remote areas where there are reduced or no interventional cardiology services to enable access to 24/7 cardiac catheterisation laboratories.

To demonstrate feasibility, Madder et al. performed remote R-PCI using the CorPath 200 platform in a series of 20 patients [40]. The operator controlled the intervention using wired R-PCI and wireless telecommunication systems (live audio and video feeds) from a room that was isolated from the procedural confines. Cath lab support was provided by a nurse and scrub technician [40]. In these patients, 19 of 22 lesions, ranging from ACC/AHA lesion class A to C, were successfully treated via remote R-PCI (technical success rate of 86.4%). In two lesions, the stent could not be advanced via R-PCI, and manual conversion with advanced interventional techniques (buddy wire, guide extension catheter) was needed [40]. In one case, the target lesion could not be crossed with a balloon either robotically or manually. Overall, procedural success was achieved in 19 of 20 patients (95.0%) [40].

In 2019, Patel et al. reported on a series of five patients with type A coronary lesions undergoing tele-R-PCI, with the first operator being 20 miles away from the procedural room [41]. For patient safety, a second in-lab interventional team was present and provided arterial access and assistance with the robotic arm preparation and device loading [41]. In this elective group of patients with ACC/AHA type A lesions, the primary endpoint (procedural success (diameter stenosis < 10%) without MACE) was met in all five patients, and the technical device success (successful completion of R-PCI, without unplanned conversion to manual assistance) rate was 100% [41].

Sooknanan et al. described the use of R-PCI in the setting of infectious disease (COVID-19), allowing the operator and cath lab staff to significantly increase their distance from the infectious patient [42].

However, the limitations are evident and clearly demonstrated in the context of higher-complexity-grade lesions, requiring escalation to complex interventional techniques or, in the case of complications, needing manual conversion. Furthermore, the applicability and safety of tele-controlled R-PCI in patients with acute coronary syndromes, where time is of the essence, is yet to be demonstrated. Last but not least, a local team is necessary, not only to first gain arterial access and load devices but also to function as a bail-out team able to convert to manual PCI and to manage procedural complications.

### 5.2. Cost-Effectiveness and Economic Impact

Although particular to national healthcare systems, the cost-effectiveness of medical procedures is an important consideration. In their propensity-score-matched analyses, Mangels et al. compared hospitalisation and resource costs for patients undergoing R-PCI to the costs for patients undergoing conventional M-PCI [43]. The total hospitalisation costs and catheterisation lab costs were comparable, whereas direct supply costs were significantly higher in patients undergoing R-PCI, with a mean difference of USD 511.32 (*p* = 0.02) [43].

Corindus Vascular Robotics was purchased by Siemens Healthineers in 2019 for about USD 1.1 billion, but due to poor adoption of the Corindus Corpath GRX platform among cardiologists and vascular interventionalists, as well as in the context of a 39% revenue fall in the company’s diagnostic branch (associated with a decreasing demand for COVID-19 antigen tests as well as other factors), Siemens Healthineers decided to discontinue the platform and transform the branch to a research and development project to further the development of the robotic platform for the field of neurovascular interventions [44].

### 5.3. Future Iterations of R-PCI Platforms

Considering the above-mentioned limitations, more advanced robotic systems allowing a wider range of motion and actions may be worth investigating. In a single proof-of-concept case, Li et al. used a novel concept multigripper robotic platform (ALLVAS, Oppen, Shanghai, China) providing four independent manipulator arms and thus allowing 12 degrees of freedom [45]. Although this approach of simulating two pairs of operator hands is conceptually interesting, this platform showed known limitations, needing manual assistance for device exchanges, balloon/stent deployment and intravascular imaging [45].

## 6. Conclusions

R-PCI has been shown to be associated with high technical and clinical success rates across different trials and R-PCI platforms in selected patients. Propensity-score-matched analyses show safety outcomes and MACE rates that are comparable to those of conventional M-PCI [4,5]. Although the applicability of R-PCI in telemedicine has been shown, this setting is limited by the necessity of a second interventional team at the patient site in case of emergencies [40,41]. R-PCI can significantly reduce the first operator’s exposure to radiation and thus allow a reduction in the feared deterministic and stochastic effects of occupational exposure to radiation [11]. Nonetheless, further investigations are needed to determine whether R-PCI effectively reduces radiation exposure for the rest of the cath lab staff. To date, we have conflicting results around patient exposure to radiation [4,5]. Current data suggest that the contrast volumes needed for patients undergoing R-PCI are comparable to those undergoing M-PCI [4,5,27,40]. The lack of adoption of R-PCI by interventional cardiologists may be explained by the fact that current generations of R-PCI platforms are limited by their incompatibility with advanced interventional devices needed for escalation in complex interventions and the treatment of chronic total coronary occlusions [4,7,21,24,25,27]. A further major limitation of R-PCI is that currently available data on efficacy and safety are based on selected cohorts of patients, and all-comer, randomised controlled trials are lacking.

## Figures and Tables

**Table 1:** Efficacy endpoints across different trials and R-PCI platforms. (CTO = chronic total occlusion; MACE = major cardiovascular event; N = no; N/A = not applicable; R-PCI = robotically assisted percutaneous coronary intervention; RNS = remote navigation system; Y = yes) [4,5,6,7,21,24,25,26,27,28,29,30,31,32,33].

Year	Authors Journal	R-PCI Platform	N R-PCI	Control Group	N Controls	Efficacy Endpoint	Results for R-PCI
2006	Beyar et al. [6] *JACC*	RNS	18	Y	20	**Technical success:** the ability to complete the procedure without reverting to manual mode **Clinical success:** the ability to successfully complete the procedure without complications	**Technical success**: 83% **Clinical success:** 100%
2013	Weisz et al. [24] *JACC*	CorPath 200	164	N	N/A	**Technical succes**s: the successful manipulation of intracoronary devices using the robotic system only **Clinical success**: <30% residual stenosis, without MACEs within 48 h or hospital discharge	**Technical success:** 98.8% **Clinical success:** 97.6%
2014	Smilowitz et al. [25] *J. Invasive Cardiol*	CorPath 200	40	Y	80	**Technical success:** the successful intracoronary advancement of intravascular devices by the robotic system without conversion to manual operation **Clinical success:** <30% residual stenosis by visual assessment at the target lesion in the absence of MACEs before discharge	**Technical success:** 95% **Clinical success:** 100%
2017	Madder et al. [26] *Cardiovasc Revasc Med*	CorPath 200	45	Y	123 + 168	**Technical success:** N/A **Clinical success:** <30% residual stenosis	**Technical success:** N/A **Clinical success:** 100%
2017	Mahmud et al. [4] *JACC Cardiovasc Interv*	CorPath 200	108	Y	226	**Technical success:** clinical success + the completion of PCI entirely robotically or with partial manual assistance (but the completion of the procedure using the re-engaged robotic drive) **Clinical success:** <30% residual stenosis, with TIMI flow grade 3, without an in-hospital MACE	**Technical success:** 91.7% a. 81.5% completely robotic b. 11.1% partial manual assistance (7.4% manual conversion, 0.9% MACE) **Clinical success:** 99.1%
2019	Walters et al. [33] *Catheter Cardiovasc Interv*	CorPath 200	103	Y	210	**Technical success:** clinical success + the completion of PCI entirely robotically or with partial manual assistance (but the completion of the procedure using the re-engaged robotic drive) **Clinical success:** <30% residual stenosis, without an in-hospital MACE	**Technical success:** N/A (no reanalysis performed; data were previously published in the CORA-PCI study—Mahmud et al. JACC 2017 [4]) clinical success: N/A
2018	Smitson et al. [7] *J Invasive Cardiol*	Corpath GRX	40	N	N/A	**Technical success:** clinical success without unplanned manual assistance or conversion to manual PCI for procedural completion **Clinical success:** <30% residual stenosis and TIMI 3 flow, in the absence of an in-hospital MACE	**Technical success:** 90.0% **Clinical success:** 97.5%
2020	Hirai et al. [27] *Catheter Cardiovasc Interv.*	CorPath GRX	49	Y	46	**Technical success:** not specified **Clinical success:** N/A; only patients with successful PCI of a single CTO lesion were included in the analysis	**Technical success:** 98% (per-protocol hybrid approach in all cases) **Clinical success:** N/A
2020	Patel et al. [5] *JACC Cardiovasc Interv.*	CorPath GRX	280	Y	280	**N/A** The primary endpoint was patient exposure to radiation, and propensity score matching was performed for a cohort of patients who underwent successful R-PCI	N/A
2021	Kagiyama et al. [29] *Heart & Vessels*	CorPath GRX	28	Y	35	**Technical success:** clinical success and the completion of the PCI entirely robotically, or with partial manual assistance **Clinical success:** <30% residual stenosis by QCA, without an in-hospital MACE (within 72 h or to hospital discharge)	**Technical success:** 90.0% **Clinical success:** 93.3%
2022	Lemos et al. [30] *Cardiovasc Diagn Ther.*	CorPath GRX	83	N	N/A	**Technical success:** <30% residual stenosis and no unplanned manual conversions **Clinical success:** angiographic success (not specified)	**Technical success:** 85.7% **Clinical success:** 99.1%
2022	Brunner et al. [31] *Heart & Vessels*	CorPath GRX	71	N	N/A	**Technical success:** the completion of PCI with no or partial manual assistance (planned and unplanned), ultimate robotic completion of the procedure **Clinical success:** angiographic success: residual diameter stenosis <20% and TIMI 3 flow	**Technical success:** 94.2% **Clinical success:** 100%
2023	Häner et al. [32] *Front. Cardiovasc. Med.*	CorPath GRX	21	N	N/A	**Technical success:** clinical success and the completion of the PCI entirely robotically, or with partial manual assistance **Clinical success:** <30% residual stenosis by visual estimation, without an in-hospital MACE	**Technical success:** 81% **Clinical success:** 100%
2024	Leung et al. [28] *Heart Lung Circ*.	CorPath GRX	21	N	N/A	**Technical success:** the completion of R-PCI without unplanned manual conversion **Clinical success:** <30% residual stenosis by QCA, without an in-hospital MACE	**Technical success:** 81% **Clinical success:** 100%
2023	Durand et al. [21] *Eurointervention*	R-One	62	N	N/A	**Technical success:** treatment of all the target lesions using the R-One system without total conversion to manual operation **Clinical success:** absence of intraprocedural complications	**Technical success:** 95.2% **Clinical success:** 100%

**Table 2:** Reasons for conversion from R-PCI to M-PCI across the different trials. (CTO = chronic total occlusion; MACE = major cardiovascular event; tPA—tissue plasminogen activator) [4,6,7,21,24,25,26,27,28,29,30,31,32].

Authors Journal	R-PCI Platform	N R-PCI	Reasons for Conversion
Beyar et al. [6] *JACC*	RNS	18	1× unsuccessful guidewire navigation, technical problems with RNS 2× system malfunction
Weisz et al. [24] *JACC*	CorPath 200	164	2× severe resistance to stent delivery (need for buddy wire, guide extension catheter)
Smilowitz et al. [25] *J. Invasive Cardiol*	CorPath 200	40	2× resistance to stent delivery
Madder et al. [26] *Cardiovasc Revasc Med*	CorPath 200	45	not reported
Mahmud et al. [4] *JACC Cardiovasc Interv*	CorPath 200	108	9× limited guidewire/catheter support 8× technical limitations 3× MACEs
Smitson et al. [7] *J Invasive Cardiol*	Corpath GRX	40	1× uncrossable CTO lesion 1× uncrossable long-segment type C lesion 1× resistance to balloon crossing, need for orbital atherectomy
Hirai et al. [27] *Catheter Cardiovasc Interv*	CorPath GRX	49	1× thrombus formation, intracoronary tPA application
Kagiyama et al. [29] *Heart & Vessels*	CorPath GRX	28	1× wiring of subintimal space 2× resistance to stent/balloon delivery
Lemos et al. [30] *Cardiovasc Diagn Ther.*	CorPath GRX	83	6× unable to reach target lesion (with guidewire, balloon or stent) 2× uncrossable lesion (guidewire, stent) 2× clinically indicated conversion 1× device malfunction 4× other reasons
Brunner et al. [31] *Heart & Vessels*	CorPath GRX	71	4× friction, unable to deliver devices or repetitive use of microcatheters 1× side-branch perforation (tortuous vessel)
Häner et al. [32] *Front. Cardiovasc. Med.*	CorPath GRX	21	2× platform limitations/poor guide catheter support 1× transient slow-flow due to prolonged exchange times 1× software error
Leung et al. [28] *Heart Lung Circ.*	CorPath GRX	21	1× unsuccessful postdilatation balloon delivery 1× resistance to stent delivery 1× cassette error 1× guide catheter disengagement
Durand et al. [21] *Eurointervention*	R-One	62	1× balloon-uncrossable lesion, guide extension catheter 1× technical fault, incorrect guidewire loading into the R-PCI cassette 1× coronary dissection (NHLBI type B)

**Table 3:** Safety endpoints across different trials and R-PCI platforms. (MACE: major cardiovascular event; M-PCI: manual PCI; N: number of participants in the respective cohort; N/A: not applicable; RNS: remote navigation system; R-PCI: robotically assisted PCI).

Year	Authors Journal	R-PCI Platform	N R-PCI	Control Group	N Controls	Safety Endpoint
2006	Beyar et al. [6] JACC	RNS	18	Y	20	**MACE, procedural, in-hospital:** 0 1 non-target vessel myocardial infarction 3 weeks post-procedure
2013	Weisz et al. [24] JACC	CorPath 200	164	N	N/A	4 patients (2.4%) with modest post-procedural myocardial biomarker elevations, meeting the criteria for non-Q-wave myocardial infarction (CK-MB > 3 times ULN, in the absence of new Q-waves)
2014	Smilowitz et al. [25] J. Invasive Cardiol	CorPath 200	40	Y	80	no adverse events or elevations in CPK (>2 times the upper limit of normal)
2017	Madder et al. [26] Cardiovasc Revasc Med	CorPath 200	45	Y	123 + 168	not reported (the primary endpoint of the trial was the comparison of operator radiation exposure in different radioprotective settings)
2017	Mahmud et al. [4] JACC Cardiovasc Interv	CorPath 200	108	Y	226	**MACE, in-hospital:** 0.9% for both groups, *p* = non-significant
2019	Walters et al. [33] Catheter Cardiovasc Interv	CorPath 200	103	Y	210	**MACE, 6 months:** R-PCI 5.8% vs. M-PCI 3.3%, *p* = 0.51 **MACE, 12 months** *(primary endpoint):* R-PCI 7.8% vs. M-PCI 8.1%, *p* = 0.92 no access-site complications (BARC III or higher)
2018	Smitson et al. [7] J Invasive Cardiol	Corpath GRX	40	N	N/A	**MACE, in-hospital:** none reported
2020	Hirai et al. [27] Catheter Cardiovasc Interv	CorPath GRX	49	Y	46	**MACE:** R-PCI 10.4%, M-PCI 13.0%, *p* = 0.67
2020	Patel et al. [5] JACC Cardiovasc Interv	CorPath GRX	280	Y	280	**MACE, 30 days:** R-PCI 2.50%, M-PCI 3.21%, *p* = 0.445
2021	Kagiyama et al. [29] Heart & Vessels	Corpath GRX	28	Y	35	**MACE, 72 h/in-hospital:** 0
2022	Lemos et al. [30] Cardiovasc Diagn Ther.	CorPath GRX	83	N	N/A	**MACE, in-hospital:** R-PCI 2.4% **MACE, 30 days:** 1.2%
2023	Häner et al. [32] Front. Cardiovasc. Med.	CorPath GRX	21	N	N/A	**MACE, in-hospital:** 0 **MACE, 12 months:** 4.8% (1 non-target vessel myocardial infarction)
2024	Bay et al. [35] Eurointervention	CorPath GRX	70	Y	210	**MACE, 12 months:** R-PCI 10.5%, M-PCI 6.5%, *p* = 0.25
2024	Leung et al. [28] Heart Lung Circ.	CorPath GRX	21	N	N/A	**MACE, in-hospital:** 0
2023	Durand et al. [21] Eurointervention	R-One	62	N	N/A	**MACE, in-hospital:** 0 **MACE, 30 days:** 0

## Supplementary Materials

The following supporting information can be downloaded at https://www.mdpi.com/article/10.3390/jcm13185537/s1: Figure S1: Decision flowchart of screening, exclusion and inclusion for the systematic review.

## References

[1] Townsend N., Kazakiewicz D., Wright F.L., Timmis A., Huculeci R., Torbica A., Gale C.P., Achenbach S., Weidinger F., Vardas P. (2022). Epidemiology of cardiovascular disease in Europe. Nat. Rev. Cardiol..

[2] Grüntzig A. (1978). Transluminal dilatation of coronary-artery stenosis. Lancet Lond. Engl..

[3] Neumann F.J., Sousa-Uva M., Ahlsson A., Alfonso F., Banning A.P., Benedetto U., Byrne R.A., Collet J.-P., Falk V., Head S.J. (2019). 2018 ESC/EACTS Guidelines on myocardial revascularization. Eur. Heart J..

[4] Mahmud E., Naghi J., Ang L., Harrison J., Behnamfar O., Pourdjabbar A., Reeves R., Patel M. (2017). Demonstration of the Safety and Feasibility of Robotically Assisted Percutaneous Coronary Intervention in Complex Coronary Lesions: Results of the CORA-PCI Study (Complex Robotically Assisted Percutaneous Coronary Intervention). JACC Cardiovasc. Interv..

[5] Patel T.M., Shah S.C., Soni Y.Y., Radadiya R.C., Patel G.A., Tiwari P.O., Pancholy S.B. (2020). Comparison of Robotic Percutaneous Coronary Intervention With Traditional Percutaneous Coronary Intervention: A Propensity Score-Matched Analysis of a Large Cohort. Circ. Cardiovasc. Interv..

[6] Beyar R., Gruberg L., Deleanu D., Roguin A., Almagor Y., Cohen S., Kumar G., Wenderow T. (2006). Remote-control percutaneous coronary interventions: Concept, validation, and first-in-humans pilot clinical trial. J. Am. Coll. Cardiol..

[7] Smitson C.C., Ang L., Pourdjabbar A., Reeves R., Patel M., Mahmud E. (2018). Safety and Feasibility of a Novel, Second-Generation Robotic-Assisted System for Percutaneous Coronary Intervention: First-in-Human Report. J. Invasive Cardiol..

[8] Jeger R.V., Farah A., Ohlow M.-A., Mangner N., Möbius-Winkler S., Leibundgut G., Weilenmann D., Wöhrle J., Richter S., Schreiber M. (2018). Drug-coated balloons for small coronary artery disease (BASKET-SMALL 2): An open-label randomised non-inferiority trial. Lancet.

[9] Buchanan G.L., Chieffo A., Mehilli J., Mikhail G.W., Mauri F., Presbitero P., Grinfeld L., Petronio A.S., Skelding K.A., Hoye A. (2012). The occupational effects of interventional cardiology: Results from the WIN for Safety survey. EuroIntervention.

[10] Heidbuchel H., Wittkampf F.H., Vano E., Ernst S., Schilling R., Picano E., Mont L., Jais P., de Bono J., ESC Scientific Document Group (2014). Practical ways to reduce radiation dose for patients and staff during device implantations and electrophysiological procedures. Europace.

[11] Venneri L., Rossi F., Botto N., Andreassi M.G., Salcone N., Emad A., Lazzeri M., Gori C., Vano E., Picano E. (2009). Cancer risk from professional exposure in staff working in cardiac catheterization laboratory: Insights from the National Research Council’s Biological Effects of Ionizing Radiation VII Report. Am. Heart J..

[12] Doody M.M., Freedman D.M., Alexander B.H., Hauptmann M., Miller J.S., Rao R.S., Mabuchi K., Ron E., Sigurdson A.J., Linet M.S. (2006). Breast cancer incidence in U. S. radiologic technologists. Cancer.

[13] Finkelstein M.M. (1998). Is brain cancer an occupational disease of cardiologists?. Can. J. Cardiol..

[14] Roguin A., Goldstein J., Bar O., Goldstein J.A. (2013). Brain and neck tumors among physicians performing interventional procedures. Am. J. Cardiol..

[15] Ciraj-Bjelac O., Rehani M.M., Sim K.H., Liew H.B., Vano E., Kleiman N.J. (2010). Risk for radiation-induced cataract for staff in interventional cardiology: Is there reason for concern?. Catheter. Cardiovasc. Interv..

[16] Beyar R., Wenderow T., Lindner D., Kumar G., Shofti R. (2005). Concept, design and pre-clinical studies for remote control percutaneous coronary interventions. EuroIntervention.

[17] Lazar J.F., Hwalek A.E. (2023). A Review of Robotic Thoracic Surgery Adoption and Future Innovations. Thorac. Surg. Clin..

[18] Schwartz J.G., Kumar U.N., Azagury D.E., Brinton T.J., Yock P.G. (2016). Needs-Based Innovation in Cardiovascular Medicine: The Stanford Biodesign Process. JACC Basic. Transl. Sci..

[19] McGloughlin E.K., Anglim P., Keogh I., Sharif F. (2018). Innovation for the future of Irish MedTech industry: Retrospective qualitative review of impact of BioInnovate Ireland’s clinical fellows. BMJ Innov..

[20] Granada J.F., Delgado J.A., Uribe M.P., Fernandez A., Blanco G., Leon M.B., Weisz G. (2011). First-in-human evaluation of a novel robotic-assisted coronary angioplasty system. JACC Cardiovasc. Interv..

[21] Durand E., Sabatier R., Smits P.C., Verheye S., Pereira B., Fajadet J. (2023). Evaluation of the R-One robotic system for percutaneous coronary intervention: The R-EVOLUTION study. EuroIntervention.

[22] Cassese S., A Byrne R., Tada T., Pinieck S., Joner M., Ibrahim T., A King L., Fusaro M., Laugwitz K.-L., Kastrati A. (2014). Incidence and predictors of restenosis after coronary stenting in 10 004 patients with surveillance angiography. Heart Br. Card. Soc..

[23] Gupta R., Malik A.H., Chan J.S.K.M., Lawrence H.D., Mehta A.B., Venkata V.S., Aedma S.K., Ranchal P., Dhaduk K., Aronow W.S.M.F. (2022). Robotic Assisted versus Manual Percutaneous Coronary Intervention—Systematic Review and Meta-Analysis. Cardiol. Rev..

[24] Weisz G., Metzger D.C., Caputo R.P., Delgado J.A., Marshall J.J., Vetrovec G.W., Reisman M., Waksman R., Granada J.F., Novack V. (2013). Safety and feasibility of robotic percutaneous coronary intervention: PRECISE (Percutaneous Robotically-Enhanced Coronary Intervention) Study. J. Am. Coll. Cardiol..

[25] Smilowitz N.R., Moses J.W., A Sosa F., Lerman B., Qureshi Y.H., E Dalton K., Privitera L.T., Canone-Weber D., Singh V., Leon M.B. (2014). Robotic-Enhanced PCI Compared to the Traditional Manual Approach. J. Invasive Cardiol..

[26] Madder R.D., VanOosterhout S., Mulder A., Elmore M., Campbell J., Borgman A., Parker J., Wohns D. (2017). Impact of robotics and a suspended lead suit on physician radiation exposure during percutaneous coronary intervention. Cardiovasc. Revascularization Med. Mol. Interv..

[27] Hirai T., Kearney K., Kataruka A., Gosch K.L., Brandt H., Nicholson W.J., Lombardi W.L., Grantham J.A., Salisbury A.C. (2020). Initial report of safety and procedure duration of robotic-assisted chronic total occlusion coronary intervention. Catheter. Cardiovasc. Interv. Off. J. Soc. Card. Angiogr. Interv..

[28] Leung J., French J., Xu J., Kachwalla H., Kaddapu K., Badie T., Mussap C., Rajaratnam R., Leung D., Lo S. (2024). Robotic Assisted Percutaneous Coronary Intervention: Initial Australian Experience. Heart Lung Circ..

[29] Kagiyama K., Mitsutake Y., Ueno T., Sakai S., Nakamura T., Yamaji K., Ishimatsu T., Sasaki M., Chibana H., Itaya N. (2021). Successful introduction of robotic-assisted percutaneous coronary intervention system into Japanese clinical practice: A first-year survey at single center. Heart Vessel..

[30] Lemos P.A., Franken M., Mariani  M., Caixeta A., Almeida B.O., Pitta F.G., Prado G.F.A., Garzon S., Ramalho F., Albuquerque G. (2022). Safety and effectiveness of introducing a robotic-assisted percutaneous coronary intervention program in a tertiary center: A prospective study. Cardiovasc. Diagn. Ther..

[31] Brunner F.J., Waldeyer C., Zengin-Sahm E., Kondziella C., Schrage B., Clemmensen P., Westermann D., Blankenberg S., Seiffert M. (2022). Establishing a robotic-assisted PCI program: Experiences at a large tertiary referral center. Heart Vessel..

[32] Häner J.D., Räber L., Moro C., Losdat S., Windecker S. (2023). Robotic-assisted percutaneous coronary intervention: Experience in Switzerland. Front. Cardiovasc. Med..

[33] Walters D., Reeves R.R., Patel M., Naghi J., Ang L., Mahmud E. (2019). Complex robotic compared to manual coronary interventions: 6- and 12-month outcomes. Catheter. Cardiovasc. Interv. Off. J. Soc. Card. Angiogr. Interv..

[34] Harrison J., Ang L., Naghi J., Behnamfar O., Pourdjabbar A., Patel M.P., Reeves R.R., Mahmud E. (2018). Robotically-assisted percutaneous coronary intervention: Reasons for partial manual assistance or manual conversion. Cardiovasc. Revascularization Med. Mol. Interv..

[35] Bay B., Kiwus L.M., Goßling A., Koester L., Blaum C., Schrage B., Clemmensen P., Blankenberg S., Waldeyer C., Seiffert M. (2024). Procedural and one-year outcomes of robotic-assisted versus manual percutaneous coronary intervention. EuroIntervention J. Eur. Collab. Work. Group. Interv. Cardiol. Eur. Soc. Cardiol..

[36] Bezerra H.G., Mehanna E., WVetrovec G., ACosta M., Weisz G. (2015). Longitudinal Geographic Miss (LGM) in Robotic Assisted Versus Manual Percutaneous Coronary Interventions. J. Intervent Cardiol..

[37] Lee J.M., Choi K.H., Song Y.B., Lee J.Y., Lee S.J., Lee S.Y., Kim S.M., Yun K.H., Cho J.Y., Kim C.J. (2023). Intravascular Imaging-Guided or Angiography-Guided Complex PCI. N. Engl. J. Med..

[38] Koeda Y., Ishida M., Sasaki K., Kikuchi S., Yamaya S., Tsuji K., Ishisone T., Goto I., Kimura T., Shimoda Y. (2023). Periprocedural and 30-day outcomes of robotic-assisted percutaneous coronary intervention used in the intravascular imaging guidance. Cardiovasc. Interv. Ther..

[39] Kimura T., Koeda Y., Ishida M., Numahata W., Yamaya S., Kikuchi S., Ishisone T., Goto I., Itoh T., Morino Y. (2023). Safety and feasibility of intravascular ultrasound-guided robotic percutaneous coronary intervention. Coron. Artery Dis..

[40] Madder R., VanOosterhout S., Jacoby M., Collins J.S., Borgman A., Mulder A., Elmore M., Campbell J., McNamara R., Wohns D. (2017). Percutaneous coronary intervention using a combination of robotics and telecommunications by an operator in a separate physical location from the patient: An early exploration into the feasibility of telestenting (the REMOTE-PCI study). EuroIntervention J. Eur. Collab. Work. Group. Interv. Cardiol. Eur. Soc. Cardiol..

[41] Patel T.M., Shah S.C., Pancholy S.B. (2019). Long Distance Tele-Robotic-Assisted Percutaneous Coronary Intervention: A Report of First-in-Human Experience. EClinicalMedicine.

[42] Sooknanan N.N., Memon S., George J.C. (2022). Robotic Percutaneous Coronary Intervention During COVID-19 Pandemic: Outcomes and Cost Effectiveness With Procedural Distancing. J. Invasive Cardiol..

[43] Mangels D., Fregoso A., Ang L., Mahmud E. (2020). Resource Utilization During Elective Robotic-Assisted Percutaneous Coronary Intervention. J. Invasive Cardiol..

[44] (2023). Siemens Calls It Quits in Robotic Heart Surgery. https://www.mddionline.com/robotics/siemens-calls-it-quits-robotic-heart-surgery.

[45] Li P., Zhang L., Li B., Chen W.-S., Guo Z.-F., Zhang B.-L. (2023). The first experience of multi-gripper robot assisted percutaneous coronary intervention in complex coronary lesions. J. Geriatr. Cardiol. JGC.

