# Supplementary material for: Features and Limitations of Robotically Assisted Percutaneous Coronary Intervention (R-PCI): A Systematic Review of R-PCI
Source: J Clin Med. 2024 Sep 19;13(18):5537. doi: 10.3390/jcm13185537 (PMC11432210; doi:10.3390/jcm13185537)
Supplement: Supplementary file 1 [file jcm-13-05537-s001.zip › jcm-3077508-supplementary.pdf]

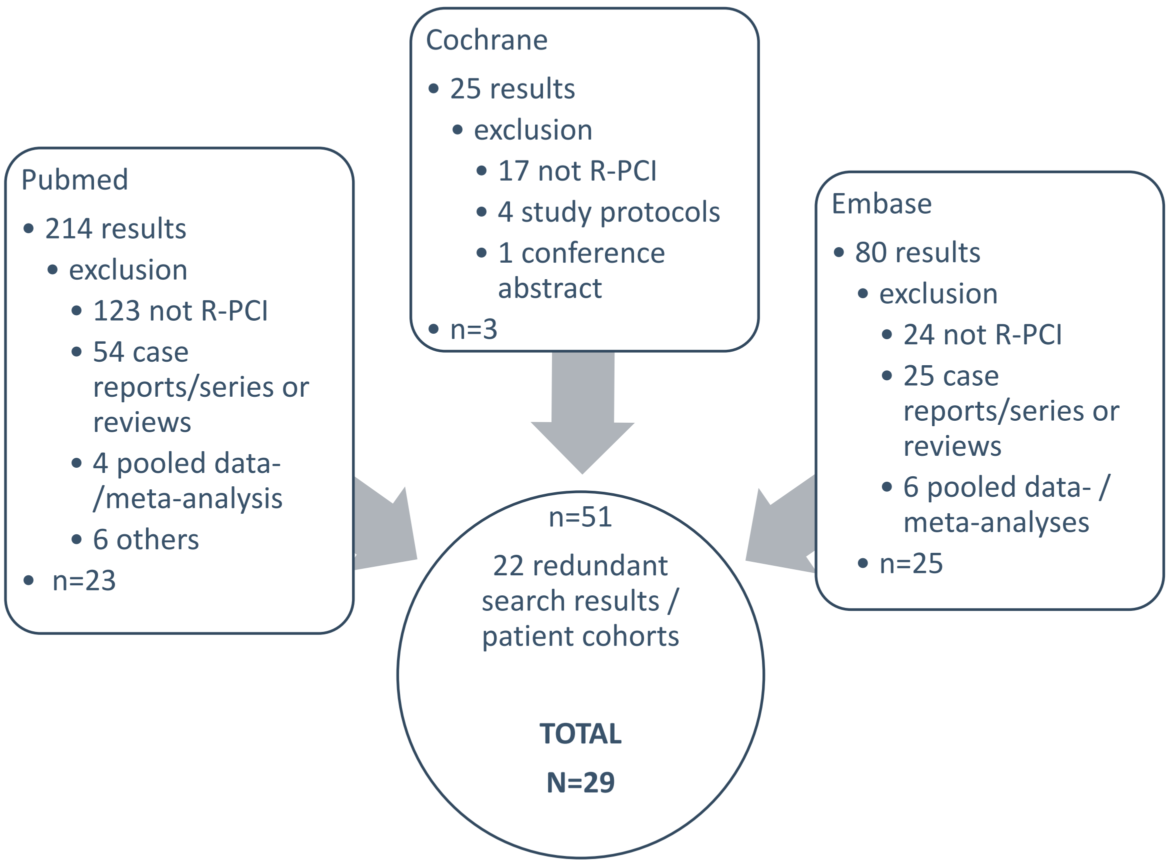

Supplementary Figure S1 – Decision flowchart of screening, exclusion and inclusion for the systematic review.
